# A 13-year real-life study on efficacy, safety and biological effects of *Vespula* venom immunotherapy

**DOI:** 10.1186/s12948-017-0079-y

**Published:** 2018-01-18

**Authors:** Marcello Albanesi, Andrea Nico, Alessandro Sinisi, Lucia Giliberti, Maria Pia Rossi, Margherita Rossini, Georgios Kourtis, Anna Simona Rucco, Filomena Loconte, Loredana Muolo, Marco Zurlo, Danilo Di Bona, Maria Filomena Caiaffa, Luigi Macchia

**Affiliations:** 1 School and Chair of Allergology and Clinical Immunology, Department of Emergency and Organ Transplantation, University of Bari-Aldo Moro, Piazza Giulio Cesare 13, Policlinico, 70124 Bari, Italy; 2 Unit of Clinical Pathology, Policlinico di Bari, Piazza Giulio Cesare 13, Policlinico, 70124 Bari, Italy; 3 School and Chair of Allergology and Clinical Immunology, Department of Medical and Surgical Sciences, University of Foggia, Via Luigi Pinto 1, 70100 Foggia, Italy

**Keywords:** *Hymenoptera* venom allergy, Allergen immunotherapy, VIT, AIT, Long-term efficacy, Venom-specific IgE, Venom-specific IgG_4_

## Abstract

**Background:**

*Hymenoptera* venom immunotherapy (VIT) is a clinically effective treatment. However, little is known about its long-term clinical efficacy and biological effects. Several mechanisms have been proposed to account for VIT efficacy, including reduction of specific IgE and induction of allergen-specific IgG_4_, but the overall picture remains elusive. We investigated *Vespula* VIT clinical efficacy up to 8 years after discontinuation and the kinetics of *Vespula*-specific IgE and IgG_4_. Out of 686 consecutive patients we retrospectively selected and analysed a series of 23 patients with *Vespula* allergy that underwent a 5-year IT course, followed by a prolonged follow-up.

**Methods:**

Clinical efficacy of VIT was assessed as number and severity of reactions to *Vespula* re-stinging events. The presence of *Vespula*-specific IgE and IgG_4_ was also monitored over time.

**Results:**

During the VIT treatment, patients were protected, reporting no reactions or mild reactions in occasion of re-stinging events. This protection was entirely maintained during the follow-up, up to 8 years. Skin reactivity (reflecting mast cell-bound *Vespula*-specific IgE) and circulating *Vespula*-specific IgE levels declined substantially during VIT. Notably, this reduction was maintained over time during the follow-up. Moreover, all the patients were analysed for IgG_4_. A robust induction of *Vespula*-specific IgG_4_ was observed during the VIT course, with a substantial decline during the follow-up.

**Conclusions:**

We conclude that *Vespula* VIT is a clinically effective treatment, which induces long-term protection after discontinuation. The reduction of specific IgE, assessed by skin tests and RAST, closely matches the VIT- induced protection, while the IgG_4_ induction seems not to be associated with VIT clinical efficacy in the long term.

**Electronic supplementary material:**

The online version of this article (10.1186/s12948-017-0079-y) contains supplementary material, which is available to authorized users.

## Background

Insect sting allergy is responsible for severe and, sometimes, life-threatening reactions. Venom immunotherapy (VIT) was proven to be effective and safe in patients with venom allergy-induced anaphylaxis [1, 2]. The clinical efficacy of VIT is commonly defined by a reduction of the severity of the allergic reactions after *Hymenoptera* stings. In clinical practice, the reduction of both skin reactivity to insect venom and specific IgE levels in serum helps corroborate the assessment of the IT clinical efficacy [3]. Indeed, taken together, these two parameters define the global levels of allergen-specific IgE, as skin reactivity is quantitatively proportional to mast cell bound IgE. Of note, the vast majority of IgE are mast cell bound, whereas serum IgE reflect the minor pool of unbound/circulating IgE.

Only some reports exist on the long-term clinical efficacy of VIT and the kinetics of bound and unbound IgE after IT discontinuation (Additional file 1: Table S1) [4–10].

In the present study, we investigated, retrospectively, the real-life long-term efficacy of *Vespula* (*Vespula* spp.) VIT and its effects of specific IgE and IgG_4_. Thus, 23 patients (18 men and 5 women), with a history of severe allergic reaction to *Vespula* sting, underwent *Vespula* VIT for 5 years, followed by an 8-year follow-up. During the study period, we monitored: (i) allergic reactions to *Vespula* stings; (ii) performed rigorously standardized quantitative skin testing; (iii) evaluated *Vespula*-specific IgE levels.

Several mechanisms have been proposed to account for VIT-induced clinical efficacy, including drop of the allergen specific IgE levels and induction of protective IgG_4_ antibodies [11–14]. In fact, IgG_4_ are considered protective antibodies for several reasons: (i) among all the IgG subtypes, they have a weak capacity to bind Fcγ Receptors and thereby a reduced ability to activate immune cells [15]; (ii) the Fc portion of IgG_4_ molecule does not fix complement, due to the low affinity for complement factor C1q [16]; (iii) IgG_4_ are functionally monovalent and unable to form immune complexes. Indeed, IgG_4_ are dynamic molecules that exchange Fab arms by swapping heavy-light chain pairs with IgG_4_ molecules of different specificities [17, 18]. This results in the production of bispecific antibodies with a substantially decreased capacity for antigen cross-linking [15, 17, 18].

Thus, we also investigated the kinetics of IgG_4_ in the 23 patients during the 5-year VIT course and the follow-up.

## Methods

### Patients

Twenty-three patients (18 men, 5 women) with severe *Vespula* allergy that underwent VIT for 5 years were retrospectively analysed in this study. These patients were monitored for allergic reactions to *Vespula* stings during 8 additional years, after VIT discontinuation.

### Inclusion criteria

We carefully analysed the clinical files of 686 patients that had access to our Hymenoptera Venom Allergy Service, from 1989 to 2010 and applied a stringent selection process based on the following criteria:

#### Diagnostic criteria

- History of severe adverse reaction to *Vespula* stinging events: only the patients that had a grade III/IV [19] reaction to a *Vespula* stinging events were included in this study.
- Clear recognition of the culprit insect in the entomological display case: only the patients that recognized clearly *Vespula* as the culprit insect were included in this study.
- Sensitization to *Vespula* venom as revealed by both skin test and RAST: patient missing either of these two parameters were excluded from this study.

#### Therapeutic criteria

Requirement of 5-year *Vespula* VIT: patients that underwent either shorter/longer VIT courses or multiple ITs for different *Hymenoptera* (e.g. *Vespula* and *Polistes*) were excluded from this study.

#### Follow-up criteria

Requirement of at least one follow-up clinical assessment (at least 3 years after VIT discontinuation), with skin tests execution and serum collection for RAST determination (see below): if one of these determinations was missing the patient was excluded from the study.

### *Vespula* venom IT

All patients had been treated with *Vespula* spp. VIT, subcutaneously (n = 13 were from ALK-Abellò VIT supplier, Milan, Italy; n = 10 were from Dome Hollister Stier Miles VIT supplier, Spokane, WA, USA). As for the ALK-Abellò VIT, the venom was purified, biologically standardized in Quality Units (SQ-U) and absorbed onto alum hydroxide gel. The maintenance dosage was 100.000 SQ-U. The amount of alum hydroxide contained in the maintenance dose was 1.35 mg. The DHS VIT was an aqueous solution of purified *Vespula* venom. The maintenance dosage was 100 μg, fully comparable with the ALK-Abellò maintenance dosage [20]. After 10–15 weeks of induction with increasing doses of *Vespula* venom, the maintenance dose was given every 6 weeks, for 5 years. Adverse reactions to the VIT injections were recorded on the clinical logbook of the patient. In particular, local reactions of less than 10 cm in diameter were considered mild local reactions. The IT protocols used are summarized in Additional file 2: Table S2 and Additional file 3: Table S3.

The insect-sting challenge test was not performed at the end of the VIT course, due to local ethical policies.

### Stings events recording

Patients were asked to recognize the stinging insect using an entomological display case.

All the patients were interviewed for re-stings and possible related adverse reactions. During VIT course, the interview process was performed every 6 weeks, at every VIT administration. During the follow-up, patients were interviewed approximately 3 and 8 years, respectively, upon VIT discontinuation.

*Vespula* re-stinging events were also recorded in the clinical logbook of the patient (along with a few occasional stings by other *Hymenoptera*). The severity of adverse reaction to stinging events was classified according to Müller [19].

### Quantitative skin testing

Skin tests were performed at baseline and 3 and 5 years after the beginning of VIT. Moreover, the tests were performed at year 3.5 ± 1.4 and 8.1 ± 3.8 after discontinuation. Skin testing was carried out in a strictly quantitative fashion by two distinct techniques: skin prick testing and intradermal testing, as described [21]. Both techniques were carried out in a single session, sequentially, in a three-step procedure. Thus, the patients were first subjected to skin prick testing, using a 100 μg/ml *Vespula* venom solution (see below) and, successively, to intradermal tests with the same allergen at two different tenfold concentrations (viz. 0.1 and 1 μg/ml, respectively).

Lyophilized *Vespula* allergen *(Vespula* spp.) was supplied by Dome–Hollister-Stier Miles, Spokane, WA, USA, and reconstituted in 1% albumin saline. Histamine hydrochloride 10 mg/ml in 50% glycerol solution (Stallergenes, Antony, France) was used as the positive control in skin prick testing. A 0.002 mg/ml aqueous solution of the same reagent was used as the positive control in intradermal testing. Saline with 1% albumin was used as the negative control in both skin prick testing and intradermal testing. Both skin prick tests and intradermal tests were performed on the volar side of the forearms.

As for skin reactivity quantitative assessment, the area of the wheals generated was calculated as described [21]. In order to achieve normalization for inter and intra-individual variations, results were expressed in terms of ratio between the *Vespula* wheal area and the homologous histamine area, referred to as Skin Index [22].

### Serum antibody measurement

Allergen-specific *(Vespula* spp.) IgE levels were measured by RAST (ImmunoCAP Thermo Fischer, Milan, Italy) in serum samples collected at baseline, approx. 3 and 6 months, respectively, from starting and, then, yearly during the VIT course. During the follow-up, serum collection took place at the same time-points as for skin testing. The sera were not diluted before the IgE assessment. Results were expressed in mass units (assuming 1U = 2.4 ng).

*Vespula*-specific IgG_4_ levels were determined in the above sera using a *Vespula* IgG_4_ ELISA kit (Dr. Fooke Laboratories, Neuss, Germany). Thus, *Vespula* pre-coated strips were used. The sera of the patients were diluted 1:101, in dilution buffer (supplied), and then added into the correspondent wells. After incubation (1 h at 37 °C) and extensive washing, 100 μl of anti-human IgG_4_-antibody conjugated with horseradish peroxidase were added, followed by 1 h incubation at 37 °C. Upon further washing, 100 μl of Substrate (also supplied by Dr. Fooke Laboratories) was added and incubated for 10 min, in the dark, at room temperature, revealing the presence of specific IgG_4_. Upon addition of 50 μl of stop solution, optical density (O.D.) measurement was carried out using a microplate reader (Biorad, model 450, Milan, Italy), at λ 450 nm. Results were expressed in mass units.

*Vespula*-specific IgG_4_ were also determined by an additional experimental approach, based on an in-house adaptation of two different commercially available antibody-revealing tools. Thus, anti-human IgG_4_ pre-coated 96-well plates (Cayman Chemical Company, Ann Arbor, USA) were used. Upon blocking with 10% non-fat dried milk, overnight, the wells were incubated at 37 °C with 50 μl of ALLERgen Basic Kit incubation buffer (RADIM, Pomezia, Italy) and 50 μl of serum of the patients, for 1 h. Subsequently, after extensive washing, biotinylated *Vespula* allergen (100 μl; also from RADIM) was added, followed by 30 min incubation at 37 °C. Upon further washing and incubation with streptavidin-conjugated horseradish peroxidase (30 min at 37 °C; RADIM) addition of the substrate (15 min at room temperature) revealed the presence of specific IgG_4_. O.D. measurement was carried out as above, at λ 450 nm.

*Vespula*-specific IgG_4_ levels were also measured in 20 adult healthy controls (14 female, 6 male; average age 32.4 ± 8.1) by the commercial kit. Only five of them were studied with the in-house technique.

### Statistical analysis

IgE and IgG_4_ changes were analysed using one-way Anova with Bonferroni post-test (N.S.: > 0.01, *p < 0.01, **p < 0.001). Error bars in figures correspond to standard error means. Moreover, the IgG_4_ results obtained in the patients were analysed against results in healthy controls, using the Student’s t Test (N.S. p > 0.05, *p < 0.05). Average and standard deviation are used in the text.

## Results

### Clinical efficacy and safety of *Vespula* VIT

Twenty-three patients were analysed in this retrospective study: 18 men and 5 women (out of a cumulative series of 686 *Hymenoptera*-allergic patients). All these patients had been diagnosed with *Vespula* venom allergy and had a clinical history of severe allergic reactions to *Vespula* stings. Moreover, they had undergone a 5-year *Vespula* VIT course, with a subsequent prolonged follow-up of up to 8 years. The clinical features of the patients at the time of diagnosis are summarized in Table 1.

**Table 1:** Clinical features of the patients enrolled in the study

Patient	Gender	Severity of reaction Pre VIT (according to Müller)	Age (years)	VIT duration (months)	Follow-up duration (months)	Total VIT dose (SQ-U ×10^6^)	Total VIT dose (mg)	VIT supplier
N.G.	M	IV	60	61	53	4.51		Alk
T.G.	M	IV	33	58	182		5.9	DHS
P.G.	M	IV	49	59	122		4.7	DHS
C.G.	F	III	21	60	184		5.1	DHS
C.G.	M	III	45	59	36		5.7	DHS
C.G.	M	III	35	60	199		6.5	DHS
D.F.T.	M	IV	51	58	103	1.82		Alk
R.F.	M	IV	44	59	151	2.05		Alk
G.G.	F	III	39	73	41	4.41		Alk
C.M.	F	IV	37	61	38	2.18		Alk
D.M.S	M	III	54	61	36	4.44		Alk
I.N.	M	IV	54	63	36	2.36		Alk
A.A.	M	III	13	63	62	2.11		Alk
C.M.	M	IV	39	64	43	2.19		Alk
D.F.	M	IV	59	64	66		5.8	DHS
T.A.	M	IV	49	62	28		5.7	DHS
V.V.	M	IV	50	59	51		5.6	DHS
T.A.	M	III	46	60	44	2.57		Alk
S.S.	F	III	29	50	36	3.85		Alk
M.S.	M	III	52	64	56	1.71		Alk
M.A.	F	III	33	64	27	2.28		Alk
V.R.	M	IV	52	62	37		6.5	DHS
M.F.	M	IV	40	64	31		5.7	DHS
Mean			42.78	61.22	72.26	5.73	2.80	
SD			11.81	4.02	55.37	0.54	1.07	

*DHS* Dome Hollister Stier, *ALK* ALK-Abellò

**Fig. 1:**
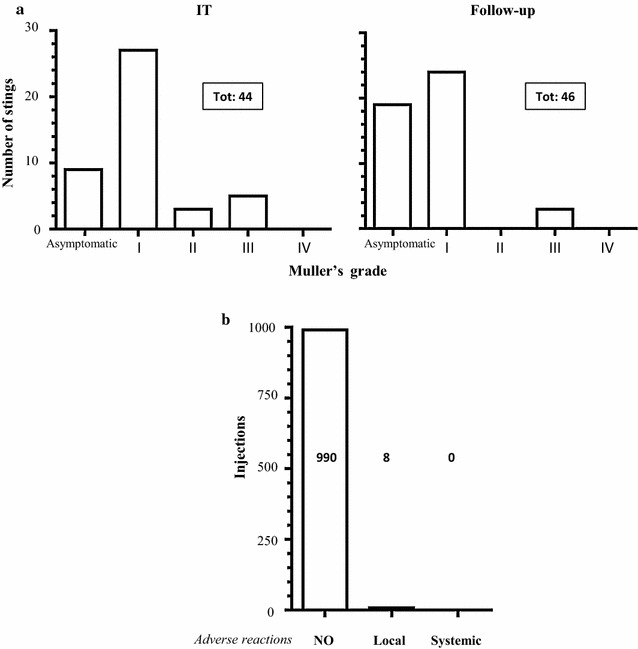
Clinical efficacy and safety of VIT. **a** Re-stinging events were counted and classified according Müller’s grade, during VIT course (left panel) and follow-up (right panel). **b** Analysis of adverse reactions to injections performed during VIT course

At first, we evaluated clinical efficacy of *Vespula* VIT. The single stinging events were assessed and the symptoms associated with their severity. During the VIT course, patients were carefully interviewed every 6 weeks, at every VIT administration. Likewise, during the follow-up, patients were personally interviewed at two different time points after VIT discontinuation (approximatively 3 and 8 years). During the VIT course, 16 patients (69.6%) were stung by a *Vespula*, with a total of 44 stinging events, evenly distributed over time (on average, 8.8 stinging events/year ± 5.4). Particularly, during the first year of VIT there were 13 stinging events in 8 patients. All the stung patients were protected. In fact, none of these events led to a major systemic reaction (grade IV according to Müller) [19], 8 events (18.2%) were followed by a mild to moderate systemic reaction (grade II–III), whereas 36 events (81.8%) were either followed by a local reaction (grade I) or were completely asymptomatic (Fig. 1a).

During the follow-up, 14 patients (60.8%) were stung by *Vespula*, with a total of 46 stings. Importantly, the vast majority of these stinging events were either followed by a local reaction or asymptomatic (43 events, 93.5%), whereas only three stings (6.5%) were followed by a moderate systemic reaction (grade III; Fig. 1a). The severity of the adverse reaction towards re-stinging events decreased over time in the same allergic individuals undergoing *Vespula* IT and subjected to multiple stings and this protection was maintained long after VIT discontinuation (data not shown).

Furthermore, we assessed the safety of VIT. To this aim, we monitored the possible adverse reactions to the subcutaneous VIT injections. During the 5-year VIT course, 998 injections in total were given. Of these, 990 (99.2%) were followed by no adverse reaction of any kinds, whereas eight injections (0.8%) were followed by a local immediate reaction. None of the injections was followed by a systemic anaphylactic reaction (Fig. 1b).

### Kinetics of *Vespula*-specific IgE

IgE interact via the Fc portion with FcεRI, expressed on different cell types, in particular mast cells. FcεRI is a high affinity receptor that has a dissociation constant (K_d_) of 10^−9^ [23]. As a consequence, the vast majority of IgE is linked to tissue resident mast cells (i.e. the bound pool). The remaining part of IgE (i.e. the much lesser unbound pool) circulates in plasma [24]. In allergic subjects, the amount of specific IgE bound on mast cell surface may be assessed through quantitative skin testing [21, 25]. The amount of unbound IgE may be evaluated by RAST [26]. Thus, using these techniques, we evaluated the kinetics of both bound and unbound *Vespula*-specific IgE, during the VIT course and the follow-up.

**Fig. 2:**
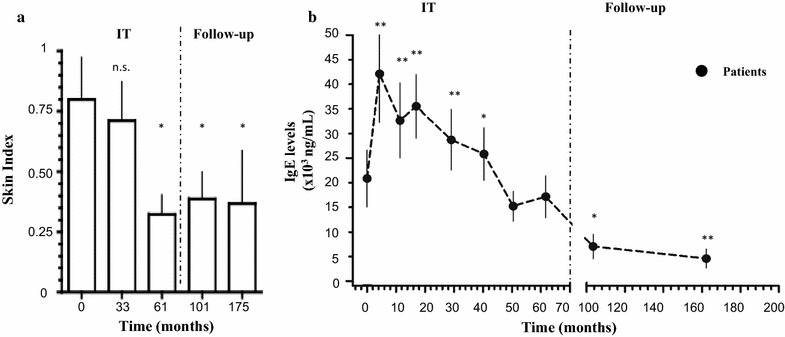
Kinetics of *Vespula*-specific IgE. **a** Kinetics of the bound pool of *Vespula*-specific IgE assessed by quantitative intradermal skin testing, at the indicated time-points (months). Skin Index represents the ratio between the area of the allergen wheal and the area of the exogenous histamine reference wheal. Intradermal skin tests were performed with a 0.1 μg/ml concentration of *Vespula* venom. Time-points on x-axis are averages of individual time-point. **b** Kinetics of circulating pool of *Vespula*-specific IgE measured using RAST, at the indicated time-points (months; averages of individual time-points). Results are expressed as mean ± SEM. Statistical significance against baseline time-point was calculated by one-way Anova with Bonferroni post-test (N.S.: > 0.01, *p < 0.01, **p < 0.001). Dashed vertical line separates the VIT course from the follow-up

As shown in Fig. 2a, during the VIT course, the skin reactivity (reflecting the bound pool of *Vespula*-specific IgE) steadily declined over time, with a 62.5% reduction after 5 years (Skin Index from 0.8 ± 0.6 to 0.3 ± 0.3). This reduction was essentially maintained throughout the follow-up.

In contrast, soon after the beginning, VIT induced a significant increase in *Vespula*-specific circulating IgE (from 20.8 × 10^3^ ± 27.0 × 10^3^ ng/ml to 42.1 × 10^3^ ± 44.8 × 10^3^ ng/ml; p < 0.001), peaking approximately 3 months after VIT initiation. After this initial transient increase, circulating IgE levels steadily declined over time, reaching an average value of 17.1 × 10^3^ ± 20.2 × 10^3^ ng/ml, at the end of the treatment, as expected [27]. During the follow-up we observed a further drop of the circulating *Vespula*-specific IgE levels, down to 4.5 × 10^3^ ± 6.1 × 10^3^ ng/ml (71.1% reduction; Fig. 2b). No differences in the kinetics of mast-cell bound and circulating IgE were observed when patients treated with ALK-Abellò and patients treated with DHS VIT were compared with each other (Additional file 4: Figure S1A).

### Kinetics of *Vespula*-specific IgG_4_

**Fig. 3:**
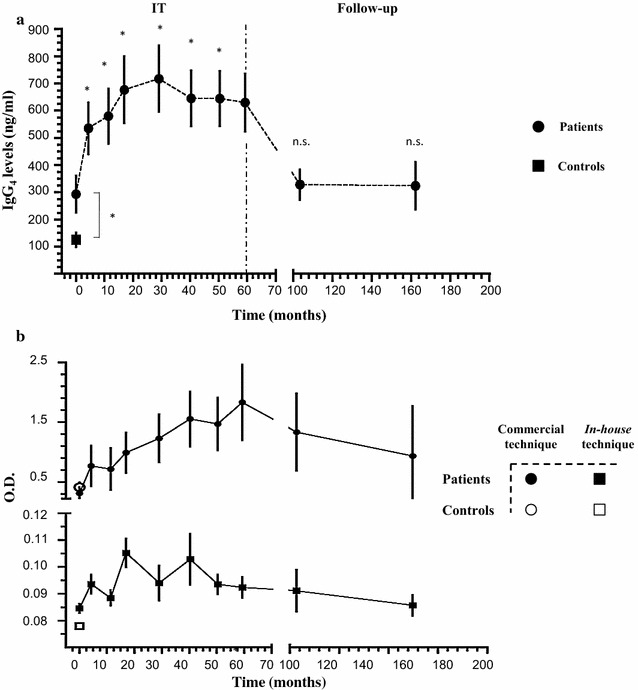
Kinetics of *Vespula*-specific IgG_4_. **a** Kinetics of serum *Vespula*-specific IgG_4_ measured using commercial ELISA kit at the indicated time-points, in months (averages of individual time-points). N = 23 patients and 20 healthy controls. Statistical significance against baseline time-point was calculated by one-way Anova with Bonferroni post-test (N.S.: > 0.01, *p < 0.01, **p < 0.001). Dashed vertical line separates the VIT course from the follow-up. **b** Comparison of measurement (O.D.) obtained with the IgG_4_ commercial kit versus the in-house technique at the indicated time-points, in months (averages of individual time-points). N = 6 patients and five healthy controls. Results are expressed as mean ± SEM

We assessed the *Vespula*-specific IgG_4_ levels, at the same time-points as for circulating IgE. Interestingly, in all these patients an increase of *Vespula*-specific IgG_4_ was detected during the VIT course (from 293.2 ± 292.8 ng/ml at baseline to 718.2 ± 527.2 ng/ml at 29 months, during the VIT course, to 630.5 ± 475.5 ng/ml at the end of the IT course). The induction of IgG_4_ was not maintained over time, during the follow-up. Indeed, a drop in the IgG_4_ levels after VIT discontinuation was observed, down to 328.3 ± 260.1 ng/ml and 323.9 ± 259.2 ng/ml at 103 and 162 months, respectively (Fig. 3a).

No differences in the kinetics *Vespula*-specific IgG_4_ were observed when patients treated with ALK-Abellò and patients treated with DHS VIT were compared with each other (Additional file 4: Figure S1B).

In order to validate the technical approach used to assess the IgG_4_ levels, we developed an in-house IgG_4_ assay, based on the adaptation of two different commercially available antibody-revealing tools. Thus, in 6 out of the 23, patients we evaluated the IgG_4_ levels using two different assays.

The set of results obtained were comparable with each other (Fig. 3b).

It is also worth noticing that *Vespula*-specific IgG_4_ levels in healthy controls at the baseline were significantly lower compared with the 23 patients.

## Discussion

It is widely accepted that *Vespula* VIT induces protection from stings in allergic patients during VIT and after discontinuation. In 2004, Golden and Colleagues monitored children with allergy to insect stings that had undergone VIT [8]. Importantly, they demonstrated that VIT in children leads to a significantly lower risk of systemic reactions to stings, up to 20 years after the treatment is stopped, suggesting that clinical efficacy is long lasting [8]. In 2008, Hafner et al. performed a long-term survey in adult patients who had previously discontinued VIT. The majority of the patients reported that the symptoms experienced with stinging events after VIT discontinuation were milder than symptoms before VIT, suggesting again long-term efficacy [9]. Moreover, Pravettoni and co-workers, analysing a series of 232 patients, found that 35.2% of the patients who could be monitored (159) reported at least one field sting up to 10 years after VIT discontinuation. None of them suffered from any systemic reactions [28].

However, few reports exists on VIT clinical efficacy long after discontinuation in adult patients (Additional file 1: Table S1) and most of them rely on data obtained through mail questionnaires sent to the patients after VIT discontinuation or telephone interviews.

Moreover, near-all these studies present some points of relative weakness, mostly because of considerable heterogeneity at different levels (Additional file 1: Table S1): (i) age of the patients, (ii) duration of the IT, (iii) type(s) of venom(s) administered during VIT (e.g. honeybee, *Vespula*) and (iv) VIT supplier used.

As for the age of patients, the outcome of the immune response is age-related [29]. Therefore, the final outcome of VIT and its long-term efficacy may vary accordingly. Moreover for duration of VIT, some of the studies on long-term efficacy included patients that underwent variable periods of VIT (from 3 to 5 years). Nonetheless, such difference might influence the outcome of the long-term immunological memory and therefore the extent of the long-term protection. Moreover, *Hymenoptera* venoms are, to some extent, cross-reactive and this could introduce biases in the assessment of VIT outcomes, when two therapies are given together. As an example, *Vespula* antigen 5 (Ves v 5), one of the major *Vespula* allergen, contains 204 amino acids residues and shares 60% of sequence identity with *Polistes dominulus* antigen 5 (Pol d 5) [30]. Therefore, patients undergoing VIT for these two different venoms could possibly develop a longer and more robust protection compared to patients receiving VIT for a single venom.

Finally, in near-all the reports present in the literature the VIT supplier is not declared. This latter point appears to be particularly relevant as *Hymenoptera* venoms comprise a complex mixture of different proteins that might all contribute to sensitization. Indeed, in vespid venom three main allergens have been described: Phospholipase A1, Hyaluronidase and Antigen 5 [31]. Importantly, the purification process of the venom extracts may vary between manufacturers [32, 33] and, therefore, the final quality and composition of the venom extracts might not be fully comparable. Thus, a different outcome in the final immune response might be obtained using different venom preparations.

In our work, we analysed a homogenous group of adult patients (mean age of 42.8 years ± 11.8) that underwent *Vespula* VIT only, for almost exactly 5 years (on average 61.2 months ± 4.0). After VIT discontinuation, we monitored all the patients over a prolonged follow-up (Table 1). Moreover, in our study, 11 patients were treated with DHS and 12 with ALK-Abellò ITs. It has to be noticed that in all the patients nearly the same induction/maintenance protocol was used (Additional file 2: Table S2 and Additional file 3: Table S3) and the final dose of venom received was fully comparable, regardless of the suppliers [20]. Interestingly, no differences were observed in terms of clinical protection in the patient treated with VIT of either of the two suppliers (data not shown). Moreover, both DHS and ALK-Abellò ITs induced comparable effects on circulating *Vespula*-specific IgE and IgG_4_ (Additional file 4: Figure S1A, B). This latter observation indirectly confirms that the immunogenicity of the VIT used was also similar.

We demonstrated that *Vespula* VIT confers a robust protection not only during the VIT course, but also long after its discontinuation. Indeed, during the follow-up, 60% of the patients were re-stung, of whom 93% were minimally symptomatic or asymptomatic and none of the patients needed to resume *Vespula* VIT, which suggests that a 5-year IT course is possibly protective and can be recommended, at least in *Vespula* allergic patients. In order to evaluate clinical efficacy of *Vespula* VIT the patients in our study were interviewed for stinging events at each single administration during VIT and at two time (at least) points during the follow-up. In the case of a sting, they were asked to recognize the culprit insect at every interview, using an entomologic display case. It has to be noticed that *Vespula* appears to be correctly recognized in 72.3% of the cases from *Vespula* allergic patients [34]. This rather stringent approach represents a novelty.

Moreover, aside clinical efficacy, we also evaluated the biological effects of *Vespula* VIT. Indeed, as shown in Additional file 1: Table S1, few reports assessed the long-term biological effects of venom IT. Even though a drop of circulating specific IgE titres was sometimes observed in up to 70% of the patients undergoing VIT [35], only little information is present in the literature on the kinetics of venom-specific IgE long after treatment discontinuation.

As it is known, IgE molecules have a unique immunological behaviour. Indeed, at the steady state, the vast majority of IgE is bound on the surface of the mast cells via the interaction with FcεRI, a high affinity receptor, able to bind a single IgE molecule with K_d_ of 10^−9^ [23]. The remaining smaller pool of IgE circulates in plasma in unbound form. Therefore, in our study, we monitored both bound and unbound pools of *Vespula*-specific IgE.

On the one hand, changes in the bound pool of IgE were assessed with skin testing, performed in a strictly quantitative fashion. Data of this kind are particularly scarce in the context of long-term studies. Indeed, skin test results might be influenced by several factors such as circadian rhythm and technical expertise of the operator performing skin testing, thus demanding methodological rigour. Moreover, in order to render our data comparable with each other, we put emphasis on achieving normalization of the results. To this aim, we expressed this parameter by a Skin Index, previously defined as the ratio between the area of the wheal generated by the *Vespula* allergen and the area of the wheal generated by exogenous histamine [21]. Remarkably, we observed a robust reduction of skin reactivity (62.5% reduction after 5 years of VIT) after VIT that was maintained throughout the follow-up (Fig. 2a). To our knowledge, few works have so far analysed skin reactivity/variation in mast cell-bound specific IgE in a strictly quantitative fashion at different time points. In particular, before VIT, during the VIT course and long after VIT discontinuation.

On the other hand, the unbound pool of *Vespula* specific IgE was assessed by RAST. In line with other reports [36, 37], we observed a transient increase of circulating *Vespula*-specific IgE early after VIT initiation, peaking at 3 months (Fig. 2b). This time-point corresponds to the end of the IT induction phase, during which the allergen is administered weekly, at increasing doses. Thus, the increase in *Vespula*-specific IgE titres might be explained by the presence in allergic subjects of memory IgE B cells. These cells are able to differentiate into IgE-producing plasma cells upon allergen encounter [38, 39]. Interestingly, during this initial phase of VIT, the patients are already protected towards re-stinging events. Indeed, during the first year of VIT course, we observed 13 re-stinging events (in 8 patients) that were either asymptomatic or followed by a reaction of grade I of the Müller scale. This observation suggests that the clinical protection is already present early during the VIT course, despite the high *Vespula*-specific IgE levels. This initial increase was followed by a progressive and steady decline during the maintenance period. Surprisingly, we observed a persistent and further reduction of circulating IgE levels long after VIT discontinuation. This latter data suggest the induction of immunological changes, possibly inducing tolerance, resulting in the decline of specific IgE levels (Even though mast-cell bound *Vespula*-specific IgE levels seemed not to decline further during the follow-up) (Fig. 2a, b).

The cross-comparison between the clinical data, the quantitative skin test analysis and the data on circulating *Vespula*-specific IgE seems to suggest that the drop of *Vespula*-specific IgE levels might be mechanistically related to the long-term efficacy of VIT. As a consequence, based on our data, one can propose to use the venom-specific IgE levels (assessed after discontinuation) as a biomarker for long-term VIT clinical efficacy, at least in *Vespula* allergy.

However, the precise immunological mechanisms underlying VIT efficacy are probably more diverse and complex and still unclear [40–43]. Particularly, VIT has been shown to induce a rise in specific IgG_4_ levels. It is well known that a regular and persistent exposure to an antigen is able to induce IgG_4_ antibodies [44]. As mentioned above, IgG_4_ should be considered anti-inflammatory antibodies. Indeed, IgG_4_ are dynamic molecule that behave as monovalent antibodies and therefore cannot form immune complexes [17]. Moreover IgG_4_, due to their poor binding capacity to both complement and FcγRs, activate only weakly Fc-dependent immune mechanisms, such as antibody dependent cytotoxicity or the complement cascade [15, 16]. However, even though IgG_4_ have been previously proposed as a contributing factor to the clinical efficacy of VIT [45, 46], no reports have analysed the changes in the IgG_4_ levels long-after VIT discontinuation, so far (Additional file 1: Table S1).

Thus, we evaluated changes in the levels of *Vespula*-specific IgG_4_ throughout VIT and follow-up in the 23 patients studied. Remarkably, we found that *Vespula*-specific IgG_4_ rose and then reached a plateau during the VIT course but declined substantially during the follow-up, since the IgG_4_ titres at 3 and 8 years follow-up time-points are comparable to the ones observed before VIT (Fig. 3a).

This latter result suggests that long-term protection induced by VIT could be IgG_4_-independent. Interestingly, a previous report by Varga et al. [47] analysed the role of honeybee specific IgG_4_ in a group of 10 children that had undergone honeybee VIT. In line with our results, the authors of this study after a 2-year follow-up found no correlation between honeybee specific IgG_4_ levels and long-term efficacy of VIT.

In our study we evaluated, for the first time, *Vespula*-specific IgG_4_ using two different technical approaches. In particular, the commercial kit for *Vespula*-specific IgG_4_ was based on the use of *Vespula* allergen pre-coated strips. In this assay, after the incubation with the patient serum, the presence of IgG_4_ is revealed using of anti-human IgG_4_-antibody conjugated with horseradish peroxidase. Nonetheless, in this experimental setting, the total amount of IgG_4_ revealed might be underestimated. Indeed, during the incubation, other *Vespula*-specific antibodies with different isotypes (e.g. IgG_2_, IgE), present in the patient serum, will most likely compete with IgG_4_ for the binding to the cognate antigen. In order to overcome this possible competition and to ascertain the results obtained with the commercial kit for *Vespula*-specific IgG_4_, we developed an *in house*-technique that we applied to six patients and nine normal controls. This assay relies on the use of pre-coated anti-IgG_4_ wells. After incubation with the patients’ sera, *Vespula*-specific IgG_4_ are revealed using biotinylated *Vespula* allergen. Remarkably, the results obtained with the two distinct technique were comparable, thus validating our findings on IgG_4_ (Fig. 3b).

It has to be noticed that IgG_4_ titres at the beginning of the VIT were already higher compared to the 20 healthy controls, suggesting that in *Hymenoptera* venom allergic subjects an IgG-driven immune response already occurs independently from VIT. To our knowledge, this is the only study in which pre-VIT values of IgG_4_ in *Vespula* allergic patients are compared to those found in a group of normal adult individuals. Finally, we analysed the trend of the ratio between the *Vespula*-specific IgE and IgG_4_ over all the study period. Interestingly, this ratio progressively declines (from 70.1 to 27.2, at the end of the VIT course). This decline appears to be even more pronounced at the end of the follow-up (14.2) (Additional file 4: Figure S1C). This latter result seems to corroborate the hypothesis that indeed *Vespula* venom VIT induces long-lasting immunological changes.

## Conclusions

**Fig. 4:**
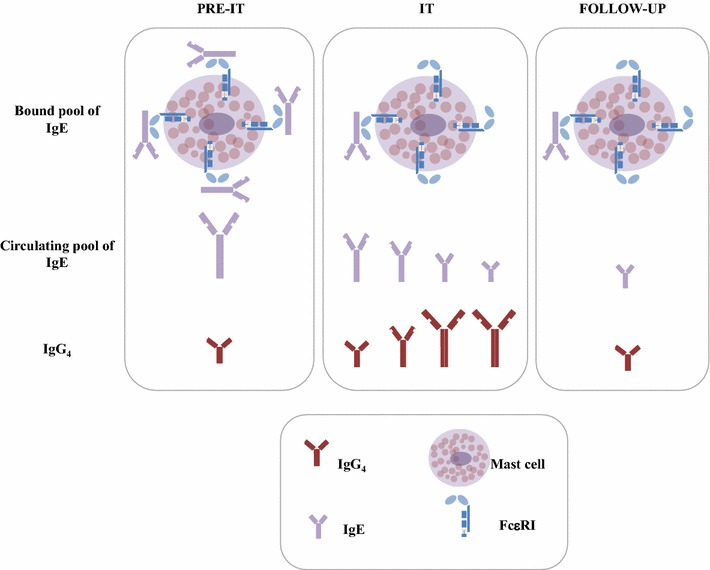
Suggested model of long-lasting immunological changes induced by VIT. Before VIT, *Vespula*-specific IgE and IgG_4_ antibodies are already present in the patients. VIT induces a progressive and steady decline in both bound and circulating pools of IgE. In contrast, IgG_4_ levels increase. The IgE levels remain low long after VIT discontinuation, while the IgG_4_ decline to pre-VIT values

In conclusion, our results obtained in a relatively small but well-controlled cohort of patients (out of a series of 686 patients) show that *Vespula* VIT induces a robust and long-lasting protection towards re-stinging events. The results obtained on *Vespula*-specific IgE levels show that IgE levels (bound and unbound) closely match the VIT clinical efficacy. Particularly, IgE levels in the follow-up, are perhaps the best predictor of clinical efficacy of *Vespula* VIT. Furthermore, IgG_4_ levels were sustained during VIT course but declined substantially after the end of VIT and, therefore, are less useful in assessing clinical efficacy of VIT in the long term. Rather, specific IgG_4_ could be used as a signature of valid immune stimulation induced by VIT (Fig. 4).

## Additional files

**Additional file 1: Table S1.** Comparative table of the existing reports on long-term clinical efficacy of VIT.

**Additional file 2: Table S2.** ALK-Abellò VIT protocol.

**Additional file 3: Table S3.** DHS VIT protocol.

**Additional file 4: Figure S1.** Kinetics of *Vespula-*specific IgE (A) and IgG4 (B) in response to DHS or ALK-Abellò VIT (C) Ratio between *Vespula*-specific IgE and IgG4 during VIT course and follow-up. Data in A and B were analised by ANOVA with Bonferroni post-test (n.s.>0.01).

## Abbreviations

VIT: venom immunotherapy
RAST: radioallergosorbentest
OD: optical density
DHS: Dome–Hollister Stier
SQ-U: Standard Quality Unit
