# Supplementary material for: A 13-year real-life study on efficacy, safety and biological effects of Vespula venom immunotherapy
Source: Clin Mol Allergy. 2018 Jan 18;16:2. doi: 10.1186/s12948-017-0079-y (PMC5774115; doi:10.1186/s12948-017-0079-y)
Supplement: Supplementary file 1 — Additional file 1: Table S1. Comparative table of the existing reports on long-term clinical efficacy of VIT. [file 12948_2017_79_MOESM1_ESM.docx]

**Study Venom sensitization VIT performed VIT Duration FU Duration Total VIT VIT IgE IgG_4_ dose supplier assessment assessment**

Keating^(4)^ honeybee honeybee 2 to 10 years 1 to 5 year not stated not stated yes (RAST) no

(1991) vespula vespula

other vespids honeybee + vespula

mixed vespids

mixed vespids +wasp

mixed vespids + honeybee

Reisman ^(10)^ not stated not stated <1 to 5 years 1 to 5 years not stated not stated no no

(1993)

Golden^(5)^ honeybee honeybee 5 to 9 years 1 to 5 years not stated not stated yes (RAST) yes*

(1996) vespula vespula

honeybee + vespula

Golden^(6)^ Multiple Multiple 5 or longer 3 years not stated not stated yes (RAST) no

(1998)

Lerch^(7)^ honeybee honeybee 3 to 5 years 1 to 6 years not stated Pharmalgen yes (RAST) yes*

(1998) vespula vespula ALK

Golden^(8)^** not stated not stated 1 to 5 years > 10 years not stated not stated no no

(2004)

Hafner^(9)^ honeybee honeybee 1 month to not stated not stated not stated no no

(2008) vespula vespula 10 years

combined honeybee + wasp

Pravettoni^(28)^ vespula vespula 5 years up to 10 years not stated not stated yes (RAST) no

(2015)

**Table S1:** comparative table of the existing reports on long-term clinical efficacy of V IT

*Total IgG levels are reported, not IgG_4_ levels. **Study performed in children.
