# Supplementary material for: A 13-year real-life study on efficacy, safety and biological effects of Vespula venom immunotherapy
Source: Clin Mol Allergy. 2018 Jan 18;16:2. doi: 10.1186/s12948-017-0079-y (PMC5774115; doi:10.1186/s12948-017-0079-y)
Supplement: Supplementary file 2 — Additional file 2: Table S2. ALK-Abellò VIT protocol. [file 12948_2017_79_MOESM2_ESM.docx]

**Week Injection Venom VIT concentration Volume administered Total VIT dose**

**(n) (n) (U/ml) (ml) (SQ-U)**

1 1 100 0.2 20

2 2 100 0.4 40

3 3 100 0.8 80

4 4 1000 0.2 200

5 5 1000 0.4 400

6 6 1000 0.8 800

7 7 10000 0.2 2000

8 8 10000 0.4 4000

9 9 10000 0.8 8000

10 10 100000 0.1 10000

11 11 100000 0.2 20000

12 12 100000 0.4 40000

13 13 100000 0.6 60000

14 14 100000 0.8 80000

15 15 100000 1.0 100000

17 16 100000 1.0 100000

21 17 100000 1.0 100000

27 18* 100000 1.0 100000

*From injection n.19 to the end of VIT course, injections were given every 6 weeks.

**Table S2:** ALK-Abellò VIT protocol.
