# Supplementary material for: A 13-year real-life study on efficacy, safety and biological effects of Vespula venom immunotherapy
Source: Clin Mol Allergy. 2018 Jan 18;16:2. doi: 10.1186/s12948-017-0079-y (PMC5774115; doi:10.1186/s12948-017-0079-y)
Supplement: Supplementary file 3 — Additional file 3: Table S3. DHS VIT protocol. [file 12948_2017_79_MOESM3_ESM.docx]

**Week Injection Venom IT concentration Volume administered Total VIT dose**

**(n) (n) (μg/ml) (ml) (μg)**

1 1 1 0.05 0.05

2 2 1 0.10 0.10

3 3 1 0.20 0.20

4 4 1 0.40 0.40

5 5 10 0.05 0.50

6 6 10 0.10 1

7 7 10 0.20 2

8 8 10 0.40 4

9 9 100 0.05 5

10 10 100 0.10 10

11 11 100 0.20 20

12 12 100 0.40 40

13 13 100 0.60 60

14 14 100 0.80 80

15 15 100 1.0 100

17 16 100 1.0 100

21 17 100 1.0 100

27 18* 100 1.0 100

*From injection n.19 to the end of VIT course, injections were given every 6 weeks.

**Table S3:** DHS VIT protocol.
