# Supplementary material for: A 13-year real-life study on efficacy, safety and biological effects of Vespula venom immunotherapy
Source: Clin Mol Allergy. 2018 Jan 18;16:2. doi: 10.1186/s12948-017-0079-y (PMC5774115; doi:10.1186/s12948-017-0079-y)
Supplement: Supplementary file 4 — Additional file 4: Figure S1. Kinetics of Vespula-specific IgE (A) and IgG4 (B) in response to DHS or ALK-Abellò VIT (C) Ratio between Vespula-specific IgE and IgG4 during VIT course and follow-up. Data in A and B were analised by ANOVA with Bonferroni post-test (n.s.>0.01). [file 12948_2017_79_MOESM4_ESM.pptx]

## Slide 1
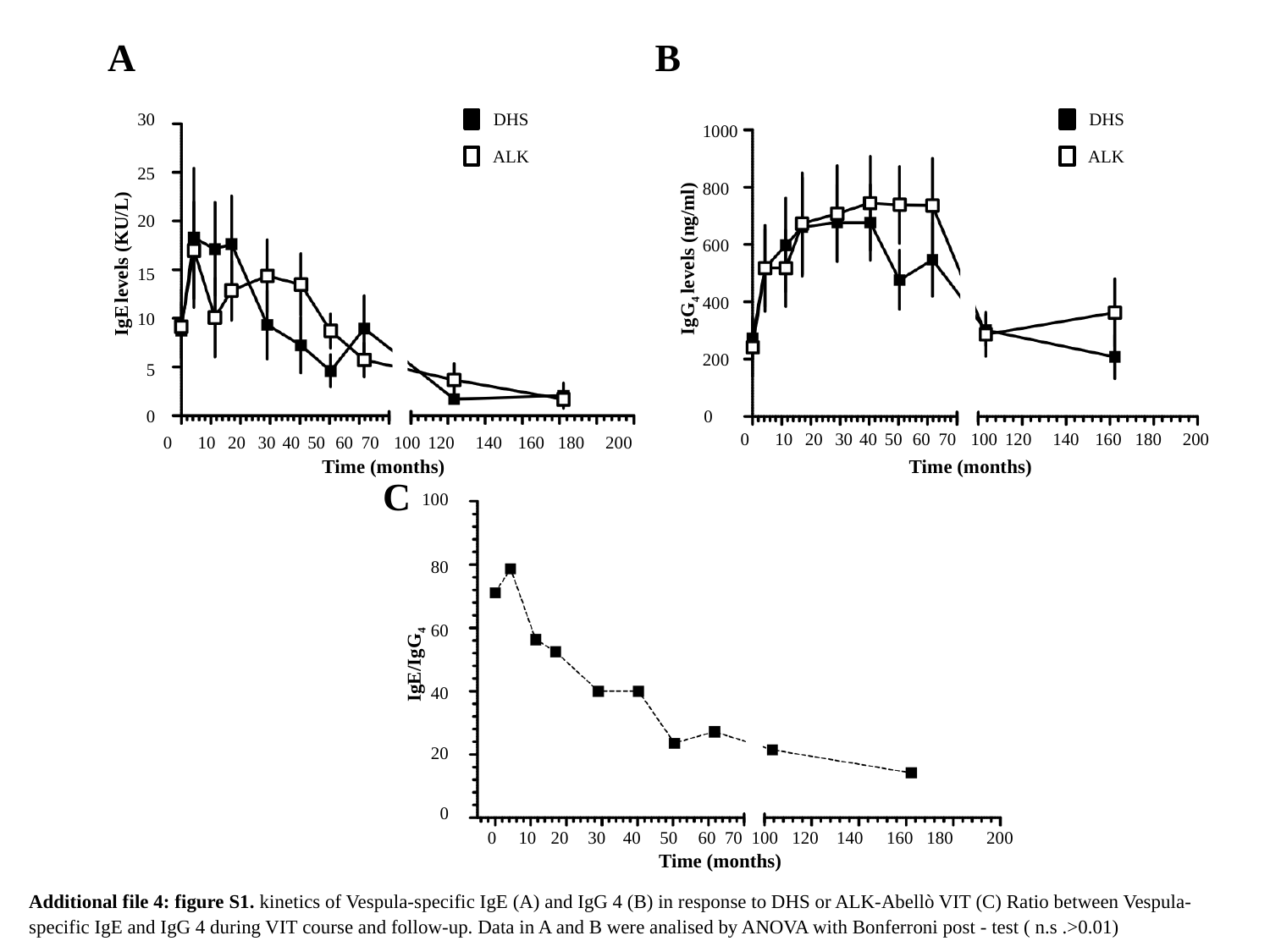

A
B
30
DHS
ALK
25
20
IgE levels (KU/L)
15
10
5
0
0
10
20
30
40
50
60
70
100
120
140
160
180
200
DHS
1000
ALK
800
600
IgG4 levels (ng/ml)
400
200
0
0
10
20
30
40
50
60
70
100
120
140
160
180
200
Time (months)
Time (months)
C
100
80
60
IgE/IgG4
40
20
0
0
10
20
30
40
50
60
70
100
120
140
160
180
200
Time (months)
Additional file 4: figure S1. kinetics of Vespula-specific IgE (A) and IgG 4 (B) in response to DHS or ALK-Abellò VIT (C) Ratio between Vespula-specific IgE and IgG 4 during VIT course and follow-up. Data in A and B were analised by ANOVA with Bonferroni post - test ( n.s .>0.01)
